# Long-term outcomes and risk factors for recurrence after lung segmentectomy

**DOI:** 10.1093/icvts/ivae125

**Published:** 2024-07-01

**Authors:** Shinsuke Uchida, Aritoshi Hattori, Mariko Fukui, Takeshi Matsunaga, Kazuya Takamochi, Kenji Suzuki

**Affiliations:** Department of General Thoracic Surgery, Juntendo University School of Medicine, Tokyo, Japan; Department of General Thoracic Surgery, Juntendo University School of Medicine, Tokyo, Japan; Department of General Thoracic Surgery, Juntendo University School of Medicine, Tokyo, Japan; Department of General Thoracic Surgery, Juntendo University School of Medicine, Tokyo, Japan; Department of General Thoracic Surgery, Juntendo University School of Medicine, Tokyo, Japan; Department of General Thoracic Surgery, Juntendo University School of Medicine, Tokyo, Japan

**Keywords:** Segmentectomy, Lung cancer, Recurrence, Long-term outcomes

## Abstract

**OBJECTIVES:**

The long-term oncological outcomes and risk factors for recurrence after lung segmentectomy are unclear. The aims of this study were to investigate the long-term prognosis and to evaluate risk factors for recurrence after segmentectomy.

**METHODS:**

Between January 2008 and December 2012, a total of 177 patients underwent segmentectomy for clinical stage I non-small cell lung cancer. The median follow-up period was 120.1 months. The overall survival (OS) and recurrence-free survival curves were analysed using the Kaplan–Meier method with a log-rank test. Univariable and multivariable analyses were used to identify significant factors that predicted recurrence.

**RESULTS:**

The study included 177 patients with a median age of 67 years. The median operative time was 155 min. No 30-day deaths were observed. Nine patients (5.1%) had recurrences: loco-regional in 3, distant in 3 and both in 3. The 5-year and 10-year recurrence-free survival rates were 89.7% and 79.8%, and the OS rates were 90.9% and 80.4%, respectively. On multivariable analysis, the risk factor associated with recurrence was a pure solid tumour [hazard ratio, 23.151; 95% confidence interval 2.575–208.178; *P *=* *0.005]. The non-pure solid tumour group had a significantly better probability of survival (5-year OS: 95.4% vs 77.2%; 10-year OS: 86.5% vs 61.8%; *P *<* *0.0001). A total of 113 patients received preoperative positron emission tomography/computed tomography. Patients with a higher maximum standardized uptake value had a significantly higher recurrence rate.

**CONCLUSIONS:**

Segmentectomy for clinical stage I non-small cell lung cancer produced acceptable long-term outcomes. Pure solid radiographic appearance was associated with recurrence and decreased survival.

## Graphical Abstract

**Figure:**
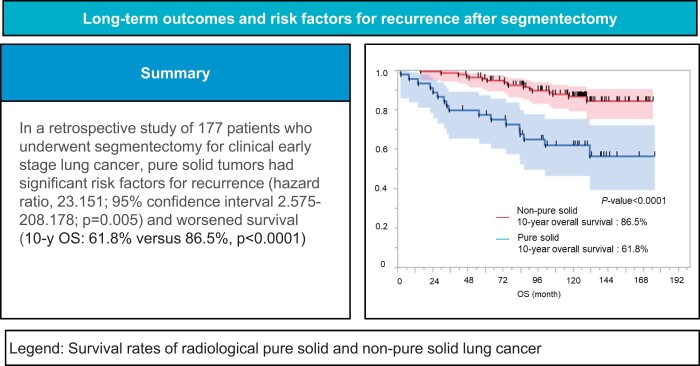

## INTRODUCTION

Computed tomography (CT) screening, including thin-section CT, has recently been used to detect small lung cancers. On pathological examination, most of the tumours with a ground-glass appearance are minimally invasive carcinomas and have a good prognosis [1]. The Japan Clinical Oncology Group (JCOG) 0201 study revealed that these patients were potential candidates for limited pulmonary resection [2]. Moreover, several reports have compared the outcomes of lobectomy and segmentectomy and shown that segmentectomy has feasible survival and recurrence rates [3, 4]. In the JCOG0802/WJOG4607L trial of patients with small-sized invasive peripheral non-small cell lung cancer (NSCLC) (≤2 cm diameter, >0.5 consolidation-to-tumour ratio; located in the outer one-third of the pulmonary parenchyma), the 5-year overall survival (OS) for patients who underwent segmentectomy was 94.3% compared with 91.1% in patients who underwent lobectomy. Although the OS was higher in the segmentectomy arm than in the lobectomy arm, recurrence-free survival (RFS) was similar between them, with a notable twofold increase in local recurrence [5]. Additionally, in the CALGB 140503 study, in patients with a peripheral tumour of 2 cm or less and pathologically negative hilar and mediastinal lymph nodes, sublobar resection was not inferior to lobectomy with respect to disease-free survival [6]. The OS mirrored that observed in both procedures, aligning with the findings of the JCOG 0802 trial. Segmentectomy is as efficacious as lobectomy for small peripheral nodules based on the results of the JCOG0802/WJOG4607L and CALGB 140503 trials [5, 6]. However, the 10-year long-term outcomes in patients who have undergone segmentectomy have not been sufficiently studied, and segmentectomy remains controversial as a viable treatment for lung cancer. Furthermore, the risk factors for recurrence after segmentectomy have not been clarified. In this retrospective study, we evaluated the long-term oncological outcomes and risk factors for recurrence in patients who underwent segmentectomy for early lung cancer at our institution.

## MATERIALS AND METHODS

### Ethical statement

A retrospective data review of surgical outcomes and patient characteristics was performed under a waiver of informed consent approved by the institutional review board of Juntendo University School of Medicine, Tokyo, Japan (institutional review board number: E23-0029).

### Eligibility

Among 1207 patients who underwent lung resection for primary lung cancer at Juntendo University Hospital between January 2008 and December 2012, a total of 177 (15%) who underwent segmentectomy for clinical stage I NSCLC were enrolled in this study. We retrospectively searched the database of our department to collect data including clinical information, baseline lung function and radiologic findings on thin-section CT scans, positron emission tomography (PET)/CT scans, surgical procedures, duration of operation, morbidity, deaths, pathological findings and short- and long-term outcomes.

### Preoperative evaluation

The clinical diagnosis was made using either preoperative bronchoscopic biopsy or thin-section high-resolution CT. Preoperative stage assignment was based on the eighth edition of the tumour, node and metastasis staging classification [7]. For clinical nodal assessment, N0 status indicated non-enlarged lymph nodes on CT. Mediastinoscopy or endobronchial ultrasound-guided transbronchial needle aspiration was not routinely performed in cases in which the mediastinal lymph nodes were not enlarged on thin-section CT or showed negative uptake of 18F-fluorodeoxyglucose on a positron emission tomography scan. The total tumour size, the size of the solid portion of the tumour and the ground-glass opacity (GGO) ratio were evaluated using CT findings. Solid appearance was defined as an area of opacification without vascular structures. The GGO was defined as a hazy density area that preserved the background vascular structures. A pure solid tumour was defined as a nodule with no GGO components.

### Operative technique

The procedure was performed by hybrid video-assisted thoracic surgery with a small skin incision [8]. Lobectomy with systemic or selective lymph node dissection is essential for patients with radiologically solid-predominant NSCLC > 2 cm. In contrast, segmentectomy tends to be used for patients with GGO-predominant tumours (<3 cm; located in the outer one-third of the pulmonary parenchyma) with limited pulmonary function and age > 80 years or the existence of multifocal GGO lesions. Tumours resected by segmentectomy should maintain an adequate margin for surgical resection. The margin between the tumour and the intersegmental plane was evaluated using a 3-direction thin-section CT scan (i.e. axial, sagittal and coronal views) prior to resection. If a sufficient surgical margin was not ensured intraoperatively, the resection line was extended to an adjacent segment of the lung. Intentional segmentectomy was defined as a case in which lobectomy was possible, with a GGO-predominant tumour or a sufficient surgical margin. If diagnosis on frozen section of the resected hilar lymph nodes was positive for malignancy, lobectomy was performed in patients with normal pulmonary function.

### Perioperative management

Segmentectomy with lymphadenectomy was performed using a hybrid thoracotomy with a minimal incision and thoracoscopy. The procedure was performed with the patient under general anaesthesia. Postoperative pain was managed with epidural anaesthesia, loco-regional nerve block or intravenous patient-controlled analgesia. Postoperative complications were graded based on the Clavien–Dindo classification system. Refractory air leakage was defined as leakage that lasted for more than 5 days and that required pleurodesis or a reoperation [9]. The chest drainage tube was removed on postoperative day 1 or 2 if the amount of drainage was < 300 ml and there was no air leakage in patients who underwent segmentectomy. Operative mortality included all deaths occurring within 30 days of the operative procedure and patients who died later but during the same hospitalization period.

### Follow-up and data collection and monitoring

After curative resection, patients were followed up by thoracic surgeons or a pulmonologist, depending on the final pathological diagnosis, with a physical examination, laboratory data with tumour markers, chest radiographs and CT scans every 6 to 12 months for at least the first 5 years. Postoperative adjuvant chemotherapy with tegafur-uracil and cisplatin plus vinorelbine was recommended for patients with stage IB and stage II or IIIA disease, respectively. If tumour markers were elevated, or if there were symptoms or clinical findings of recurrence, further evaluation, including positron emission tomography or brain magnetic resonance imaging, was performed to assess loco-regional or distant recurrence. Loco-regional recurrence was defined as a recurrence within the ipsilateral residual lobe, hilum or mediastinal lymph nodes. Histologic evaluation was performed for loco-regional tumour recurrence. Distant recurrence was diagnosed on radiologic findings (PET/CT and brain MRI). We retrospectively obtained follow-up information from the patients’ electronic medical records and through telephone interviews and questionnaires.

### Statistical analyses

The statistical analyses were performed using JMP16 (SAS Institute Inc., Cary, NC, USA). Descriptive statistics for categorical variables are reported as frequency and percentage, whereas continuous variables are reported as mean ± standard deviation or median (range), as appropriate. For categorical variables, comparisons between groups were performed using the χ^2^ test or Fisher’s exact test. Continuous variables were compared using the unpaired *t-*test or the Mann–Whitney *U* test. To identify the risk factors for recurrence, we first performed univariable logistic regression analysis between the cohorts of patients with and without recurrence. Significant risk factors for recurrence based on variables with statistically significant factors were included in the multivariable logistic regression model. Hazard ratios and corresponding 95% confidence intervals were estimated for each explanatory variable. Furthermore, OS was defined as the time interval from the date of tumour resection to the date of death from any cause or the censored date. Moreover, RFS was defined as the time interval from the date of tumour resection to the date the recurrence was detected. Survival rates were estimated using the Kaplan–Meier survival analysis. Differences in survival rates were compared using log-rank tests. Statistical significance was set at *P *<* *0.05.

## RESULTS

Between January 2008 and December 2012, pulmonary resection for primary lung cancer was performed on 1207 patients. Of these, 177 patients who underwent radical segmentectomy for clinical stage I NSCLC were enrolled. The enrolled patients comprised 103 women and 74 men, with a median age of 67 years (range, 27–88 years). The preoperative clinical stage was IA1 in 71 (40%), IA2 in 77 (44%), IA3 in 16 (9%) and IB in 13 (7%) patients. The patient characteristics are shown in Table 1.

**Table 1:** Characteristics of eligible patients (*n* = 177)

Characteristic	Number of patients (*n* = 177)	%
Age, year, median (range)	67 (27–88)	
Sex		
Male	74	42
Female	103	58
Pack-year smoking, mean ± SD	17.37 ± 29.18	
CEA, ng/ml, median (range)	2.1 (0.4–39.2)	
Clinical maximum tumour diameter, mm, median (range)	15 (5–46)	
Primary site		
Right	86	49
Left	91	51
Baseline lung function		
FVC (% predicted)	99.50 ± 16.90	
FEV_1_, (l)	2.24 ± 0.67	
FEV_1_, (% predicted)	98.14 ± 16.80	
FEV_1_/FVC, (%)	75.81 ± 8.33	
Clinical stage		
IA1	71	40
IA2	77	44
IA3	16	9
IB	13	7

CEA: carcinoembryonic antigen; FEV: forced expiratory volume; FVC: forced vital capacity; SD: standard deviation.

### Operative findings

Of 177 patients, 86 (49%) underwent a right segmentectomy. Ninety-one (51%) patients underwent a left segmentectomy. Thirty-one (18%) patients underwent a diagnostic wedge resection, and 2 (1%) patients underwent an intraoperative needle biopsy before segmentectomy. An intentional segmentectomy was performed in 141 (80%) patients. The median operative time was 155 min (range: 61–315 min). The median blood loss was 15 ml (range: 0–520 min) (Table 2).

**Table 2:** Details of the surgical procedures and outcomes

Characteristic	Number of patients	%
Right		
S1 segmentectomy	15	9
S2 segmentectomy	18	10
S3 segmentectomy	9	5
S6 segmentectomy	29	17
S8 segmentectomy	9	5
Basal segmentectomy	2	1
Other segmentectomy	4	2
Left		
Upper tri-segmentectomy	45	25
S1 + 2 segmentectomy	3	2
Lingular segmentectomy	12	7
S6 segmentectomy	24	13
Basal segmentectomy	3	2
Other segmentectomy	4	2
Simple segmentectomy	110	62
Complex segmentectomy	67	38
Intentional segmentectomy	141	80
Mediastinal lymph node dissection	48	27
Number of lymph nodes dissected	5.81 ± 6.42	
Operating time (min), median (range)	155 (61–315)	
Blood loss (ml), median (range)	15 (0–520)	
Length of postoperative stay (days), median (range)	5 (3–183)	
Postoperative complications (≥CD grade IIIa)	19	11
30-day mortality	0	0
90-day mortality	1	0.5

CD: Clavien–Dindo classification.

### Postoperative outcomes and pathologic findings

The median length of hospital stay from operation to discharge was 5 days (range: 3–183 days). Major postoperative complications of Clavien–Dindo classification grades IIIa to V were observed in 19 (11%) patients. Among these, 18 had refractory air leakage. The 30-day mortality rate was 0%. One patient died of lung cancer on postoperative day 67 with incomplete resection of multiple lymph node metastases (Table 2). The pathological nodal status was pN0 in 171 (97%) patients, pN1 in 2 (1%) and pN2 in 4 (2%). Histologic findings showed adenocarcinoma in 163 patients (92%), squamous cell carcinoma in 11 (6%) and atypical adenomatous hyperplasia in 3 (2%). The histopathological findings are summarized in Table 3.

**Table 3:** Details of the histopathological findings

Characteristic	Number of patients	%
Histologic diagnosis		
Adenocarcinoma	163	92
Squamous cell carcinoma	11	6
Atypical adenomatous hyperplasia	3	2
Pathological stage		
0	4	2
IA1	64	36
IA2	78	44
IA3	16	9
IB	7	4
IIB	2	1
IIIA	5	3
IVA	1	1

### Follow-up, survival and prognostic factors

**Figure 1:**
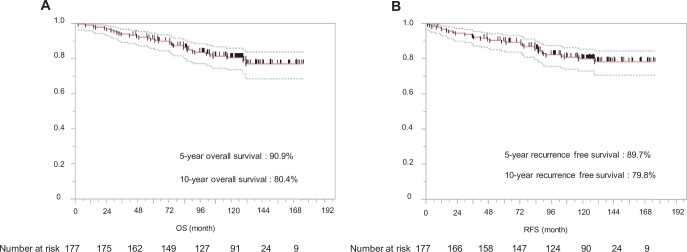
Kaplan–Meier estimates of overall survival (**A**) and recurrence-free survival (**B**) in enrolled patients. OS: overall survival; RFS: recurrence-free survival.

**Figure 2:**
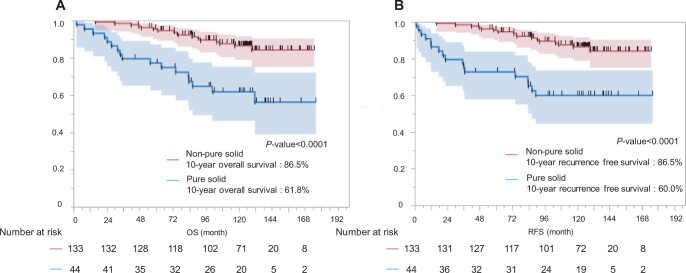
The non-pure solid group had a significantly better survival rate than the pure solid group (5-year overall survival: 95.4% vs 77.2%, 10-year overall survival: 86.5% vs 61.8%; *P *<* *0.0001) (**A**). The non-pure solid group had better recurrence-free survival than the pure solid group. (5-year recurrence-free survival: 95.4% vs 72.7%, 10-year recurrence-free survival: 86.5% vs 60.0%; *P *<* *0.0001) (**B**). OS: overall survival; RFS: recurrence-free survival.

After a median follow-up of 120.1 months (range: 2.1–176.1 months), 9 (5.1%) patients experienced recurrence. The tumour recurrences were as follows: distant 3, loco-regional 3 and both, 3. Only 1 patient had a local recurrence at the surgical margin. The recurrence group had higher carcinoembryonic antigen levels, pure solid tumours, larger tumour diameters and more advanced pathological stages. In the recurrence group, the most common primary tumour sites were the left segment 1 + 2 (*n* = 3); however, this difference was not significant. Table 4 summarizes the details of the patients with or without recurrence. The characteristics of patients with recurrence are shown in Supplementary Material, Table S1. Of the 9 patients who experienced recurrence, 6 (67%) experienced recurrence <2 years after resection. Three patients (33%) experienced recurrence more than 3 years after resection. Time to recurrence after resection is shown in Supplementary Material, Fig. S1. The OS rates at 5 and 10 years postoperatively for the entire population (*n* = 177) were 90.9% and 80.4%, respectively (Fig. 1A). The 5-year RFS and 10-year RFS rates for the entire population were 89.7% and 78.8%, respectively (Fig. 1B). At the end of the study period, 34 (19%) had died, with lung cancer accounting for 13 (7%) deaths. The risk factors for recurrence in univariable analysis were higher carcinoembryonic antigen level, pure solid tumour, maximum tumour diameter on clinical findings and maximum SUV (Table 5). In multivariable analysis, the risk factor associated with recurrence was a pure solid tumour (hazard ratio, 23.151; 95% confidence interval 2.575–208.178; *P *=* *0.005) (Table 5). Furthermore, the non-pure solid group had a significantly better survival rate than the pure solid group (5-year OS 95.4% vs 77.2%; 10-year OS 86.5% vs 61.8%; *P *<* *0.0001) (Fig. 2). Before the procedure, 113 patients underwent PET/CT. The maximum standardized uptake value (SUV max) was significantly higher in patients with recurrence. We examined the relationship between cases with or without recurrence and non-recurrence using a cut-off value of 1.7 calculated from the receiver operating characteristic curve (Supplementary Material, Fig. S2). The SUV max ≥ 1.7 group had significantly worsened OS (5-year OS: 76.2% vs 97.3% and 10-year OS: 64.0% vs 95.9%, *P *<* *0.0001) (Supplementary Material, Fig. S3).

**Table 4:** Details of the patients with and without recurrence

	With recurrence *n* = 9, (%)	Without recurrence *n* = 168, (%)	*P*-value
Age, median (range)	66.2 ± 3.5	65.5 ± 0.8	0.8526
Sex, man	6 (67)	68 (40)	0.1207
CEA, ng/ml	7.6 ± 11.2	2.9 ± 0.3	0.0048
Squamous cell carcinoma	1 (11)	10 (6)	0.5323
Radiologic pure solid tumour	8 (89)	36 (21)	<0.0001
Clinically maximum tumour diameter, mm, mean ± SD	21.8 ± 2.5	15.9 ± 0.6	0.0242
SUVmax (*n* = 113)	3.7 (1.7–16.5)	0.9 (0–19)	0.0008
Nodal metastasis	4 (44)	2 (1)	<0.0001
Pathological stage			<0.0001
0	0	4 (2)	
IA	5 (55)	153 (91)	
IB	0	7 (4)	
IIB	1 (11)	1 (1)	
IIIA/IVA	3 (33)	3 (2)	
Left	5 (56)	86 (51)	0.9378
Primary site			
Right S1	0	15 (9)	0.3488
Right S2	2 (22)	16 (10)	0.2195
Right S3	0	9 (5)	0.4760
Right S6	2 (22)	27 (16)	0.6272
Right S8	0	9 (5)	0.4760
Left upper tri-segment	3 (33)	42 (25)	0.5759
Left S6	1 (11)	24 (14)	0.7899

CEA: carcinoembryonic antigen; SD: standard deviation; SUVmax: maximum standardized uptake value.

**Table 5:** Univariable and multivariable analyses of the clinical predictors of recurrence

Variables	Univariable		Multivariable	
	HR (95% CI)	*P*-value	HR (95% CI)	*P*-value
Age (year)	1.463 (0.027–78.930)	0.8516		
Sex (male)	2.941 (0.711–12.165)	0.1364		
CEA (ng/ml)	1.085 (1.010–1.166)	0.0243	1.029 (0.953–1.112)	0.4754
Side, left	1.206 (0.313–4.648)	0.7857		
Pure solid tumour	29.333 (3.552–242.255)	0.0017	23.151 (2.575–208.178)	0.0050
Clinically maximum tumour diameter, mm	1.083 (1.007–1.165)	0.0320	1.012 (0.931–1.101)	0.7768
Maximum standard uptake value	1.231 (1.055–1.437)	0.0084		
Squamous cell carcinoma	1.975 (0.224–17.382)	0.5397		

CEA: carcinoembryonic antigen; CI: confidence interval; HR: hazard ratio.

## DISCUSSION

Radical lobectomy was reported as the standard treatment for lung cancer in 1960 [10]. A randomized controlled trial comparing lobectomy with sublobar resection in the context of OS showed that sublobar resection was associated with higher mortality and local relapse [11]. Lobectomy is the primary surgical treatment for early-stage NSCLC, whereas segmentectomy is an acceptable alternative resection method for radiographically early lung cancer in patients with GGO or older patients with low pulmonary function [1]. According to the 2018 Japanese Association of Thoracic Surgeons report, segmentectomy was performed in 5236 (11.4%) of 44 859 cases during that year [12]. Recently, the number of cases of segmental resection has increased. The JCOG0802/WJOG4607L trial concluded that segmentectomy was superior to lobectomy in terms of 5-year OS for early-stage lung cancer [13]. In the CALGB 140503 study, sublobar resection was not inferior to lobectomy with respect to disease-free survival [14]. However, long-term outcomes of ∼10 years after segmentectomy remain unclear. The morbidity and mortality rates associated with segmentectomy have been reported to be 9–32.9% and 0–1.1%, respectively, in previous studies [3, 4, 13–15] (Supplementary Material, Table S2). Major postoperative complications occurred in 19 (11%) patients at our institution. The 30-day mortality rate was 0%. These relatively low morbidity and mortality rates reflect the safety of segmentectomy in our hospital. The 5-year survival rate (90.9%) of the entire population (*n* = 177) in this study was higher than that reported in other studies (43–96%) [3, 4, 13–18] (Supplementary Material, Table S2). Furthermore, the 10-year OS rate in all patients was 80.4%, which was better than that reported in other studies (47–84%) [16, 18, 19]. There were significant differences in OS and RFS by clinical stage (Supplementary Material, Fig. S4). In our study, 34 (19%) patients died. Of these, 13 (7%) died of lung cancer and 21 (12%) died of other diseases. Of the patients who died of other diseases, 4 died of other cancers and 3 died of pneumonia. The rate of death of other diseases is high, and it is important to closely monitor patients for other diseases during follow-up. The impact of lobectomy and segmentectomy on non-lung cancer deaths depends on the extent of the patients’ comorbidities [20].

There are few reports on the 10-year long-term recurrence rate after segmentectomy. According to a report by Nishio *et al*. [16], local recurrence often occurs after right upper (21.9%) or basal segmentectomy (20.8%). In our study, recurrence was common in cases of primary lung cancer located in the left segment 1 + 2 (*n* = 3), next to the right segment 2 (*n* = 2) and right segment 6 (*n* = 2), but there was no significant difference between the segments. In this study, the 5-year and 10-year recurrence-free survival rates were 89.7% and 79.8%, respectively. These rates are better than those reported previously (65.7–94.1%, 44.0–91.0%, respectively) [16, 18, 19]. Nine (5.1%) patients experienced recurrence after segmentectomy at our institution. Among these, only 1 case recurred at the surgical margin. Of the 9 patients, 6 relapsed within 2 years and 3 relapsed beyond 3 years. Follow-up for 5 years after segmentectomy should be considered. In univariable and multivariable analyses, a pure solid tumour on preoperative CT was a significant predictor of recurrence. In the JCOG0201 trial [2], a predominantly solid tumour on CT findings had a greater rate of lymph node metastasis. The presence of a GGO component as a radiologic finding is associated with a better prognosis in non-small cell lung cancer [21–24]. Segmentectomy showed oncological results similar to those of lobectomy in solid-predominant NSCLC with a GGO component > 2–3 cm maximum tumour size [5-year OS; lobectomy (*n* = 169), 90.2%; segmentectomy (*n* = 46), 95.5%; *P* = 0.697] [25]. There was no significant difference about the prognosis comparing segmentectomy versus lobectomy in the same period of our study population with radiologic pure solid tumours. However, pure solid tumours have a potentially poor prognosis due to their pathological features [7]. According to a previous report from our department, segmentectomy was significantly associated with a higher loco-regional cancer recurrence rate (35.5% vs 15.8%; *P* = 0.001) and worse OS (59.7% vs 72.9%; *P* = 0.007) than lobectomy in the study population with T1c radiologic pure-solid NSCLC [26]. Furthermore, tumours sized > 2.0 cm were significantly associated with nodal metastasis in clinical stage IA squamous cell carcinoma [27]. Our singular case of recurrence following segmentectomy for squamous cell carcinoma involved a tumour size exceeding 2.0 cm. Tumour metabolism (≥ 1.7 SUV max) was associated with recurrence and shorter survival in subgroup analysis. The key finding in our study, a radiologic pure solid appearance on preoperative CT images, was associated with a higher risk of recurrence and worse prognosis after segmentectomy.

### Limitations

This study has some limitations. First, it was retrospectively conducted at a single institution using a small cohort of patients. Additionally, a limited number of patients underwent preoperative PET/CT to reach statistically significant conclusions. Second, the duration of post-procedure follow-up was limited. Moreover, the study focused on a specific group of patients who underwent segmentectomy without comparison to those who underwent lobectomy. Third, the number of patients experiencing recurrence was small, and drawing a conclusion from 9 recurrences is limited. However, this study has the advantage of being a single-institution study characterized by consistent surgical approaches, uniform patient selection criteria and long-term follow-up.

## CONCLUSION

Segmentectomy is associated with acceptable morbidity and mortality rates. Thus, segmentectomy can achieve feasible long-term oncological outcomes in patients with stage I NSCLC. However, a pure solid tumour on preoperative CT was a significant risk factor for recurrence, with a worse prognosis. Further prospective trials are needed to clarify the indications for segmentectomy.

## Supplementary Material

ivae125_Supplementary_Data

## Data Availability

The data underlying this article will be shared upon reasonable request from the corresponding authors.

## Notes

Presented at the IASLC 2022 World Conference on Lung Cancer, Vienna, Austria, 6–9 August 2022.

## ABBREVIATIONS

CT: Computed tomography
GGO: Ground-glass opacity
JCOG: Japan Clinical Oncology Group
NSCLC: Non-small cell lung cancer
OS: Overall survival
PET: Positron emission tomography
RFS: Recurrence-free survival
