# Supplementary material for: Long-term outcomes and risk factors for recurrence after lung segmentectomy
Source: Interdiscip Cardiovasc Thorac Surg. 2024 Jul 1;39(1):ivae125. doi: 10.1093/icvts/ivae125 (PMC11245319; doi:10.1093/icvts/ivae125)
Supplement: ivae125_Supplementary_Data [file ivae125_supplementary_data.zip › Supplementary Fig3.pdf]

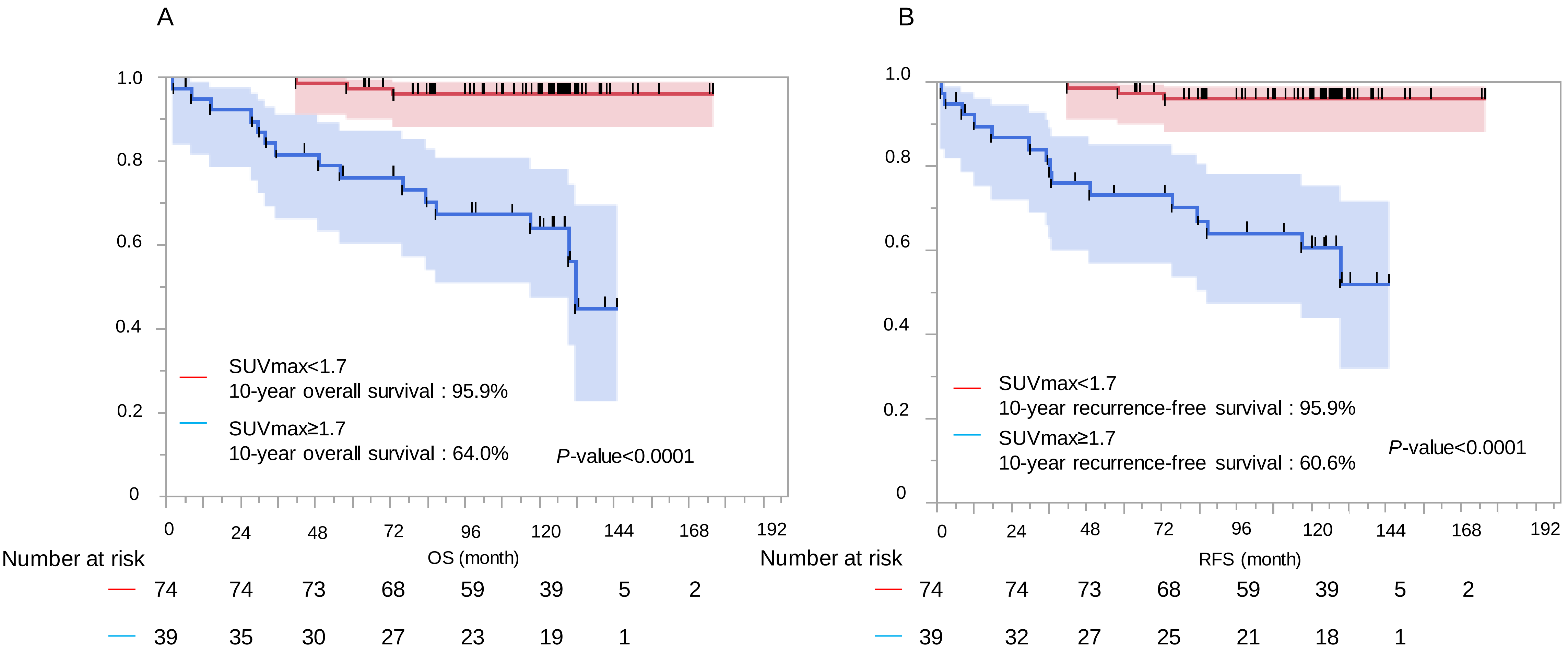

Supplementary Figure 3. The SUV max ≥1.7 group had a significantly lower survival rate (5-y OS: 76.2% versus 97.3%, 10-y OS: 64.0% versus 95.9%, P<0.0001) (A). The SUV max ≥1.7 group had a lower RFS (5-y RFS: 73.2% versus 97.3%, 10-y RFS: 60.6% versus 95.9%, P<0.0001) (B).
