# Supplementary material for: Long-term outcomes and risk factors for recurrence after lung segmentectomy
Source: Interdiscip Cardiovasc Thorac Surg. 2024 Jul 1;39(1):ivae125. doi: 10.1093/icvts/ivae125 (PMC11245319; doi:10.1093/icvts/ivae125)
Supplement: ivae125_Supplementary_Data [file ivae125_supplementary_data.zip › Supplementary Table1.docx]

| Age | Sex | Primary site | LN dissection | Histology  p-Stage | Recurrence | Recurrence site |
| --- | --- | --- | --- | --- | --- | --- |
| 74 | Woman | Right S6 | Hilum | Ad  T2aN1M0 IIB | Both | Margin Subclavian LN  Brain |
| 72 | Man | Right S6 | MLN | Ad  T1aN0M0 IA1 | Both | MLN  Brain |
| 43 | Man | Left S1+2 | MLN | Ad  T1bN2M0 IIIA | Both | MLN  Right lung |
| 80 | Man | Left S1+2 | Hilum | Ad  T1cN0M0 IA3 | Distant | Brain |
| 57 | Man | Right S2 | MLN | Ad  T1bN0M0 IA2 | Loco-regional | Right lung |
| 61 | Man | Left S6 | MLN | Ad  T2aN2M0 IIIA | Loco-regional | MLN |
| 81 | Man | Right S2 | Hilum | Sq  T1cN0M0 IA3 | Loco-regional | MLN |
| 55 | Woman | Left S8 | MLN | Ad  T1aN0M0 IA1 | Distant | Brain  Bone |
| 73 | Woman | Left S1+2 | MLN | Ad  T1cN2M0 IIIA | Distant | Brain |

**Supplementary Table 1.** Characteristics of patients with recurrence after segmentectomy

MLN; mediastinal lymph node
