# Supplementary material for: Long-term outcomes and risk factors for recurrence after lung segmentectomy
Source: Interdiscip Cardiovasc Thorac Surg. 2024 Jul 1;39(1):ivae125. doi: 10.1093/icvts/ivae125 (PMC11245319; doi:10.1093/icvts/ivae125)
Supplement: ivae125_Supplementary_Data [file ivae125_supplementary_data.zip › Supplementary Table2.docx]

**Supplementary Table 2.** Previous reports of long-term outcomes after segmentectomy for lung cancer

| Author | Year | Number (Female/Male) | Morbidity (%) | Mortality (%) | Recurrence  (%) | Local  recurrence rate  (%) | 5yrs-RFS  (%) | 5yrs-OS  (%) | 10yrs-RFS  (%) | 10yrs-OS  (%) |
| --- | --- | --- | --- | --- | --- | --- | --- | --- | --- | --- |
| Sugi | 2010 | 159  (96/63) | 9 | 0 | 11.9 | 6.3 | 94.2 | 87.3-93.3 | NR | NR |
| Okada | 2012 | 102  (52/50) | 9.8 | 0 | 11.8 | 4.9 | 84.7 | 89.8 | NR | NR |
| Nishio | 2016 | 118  (43/75) | NR | NR | 27.1 | 19.5 | 75.4 | 86.4 | 61.8 | 70.0 |
| Schuchert | 2019 | 384 | 32.9 | 1 | 20.3 | 5.7 | 69.4 | 49.9 | NR | NR |
| Nomori | 2019 | 179  (92/87) | NR | NR | 8 | 4.5 | NR | NR | 91 | 84 |
| Nguyen | 2019 | 71 (RATS)  (35/36) | 29 | 0 | 19 | 0 | NR | 43 | NR | NR |
| Onaitis | 2020 | 1654  (977/677) | NR | 1.1 | NR | NR | NR | 65.3 | NR | NR |
| Handa | 2021 | 240  (124/116) | NR | NR | 2.9 | 1.6 | 95.5 | 96.0 | NR | NR |
| Jones | 2021 | 416  (273/143) | NR | NR | NR | NR | 65.7 | 73.1 | 44.0 | 47.0 |
| This study | 2023 | 177  (103/74) | 11 | 0 | 5.1 | 1.7 | 89.7 | 90.9 | 79.8 | 80.4 |

NR, not reported; RATS, robot-assisted thoracic surgery; RFS, recurrence-free survival; OS, overall survival
