# Supplementary figures and images for: Long-term outcomes and risk factors for recurrence after lung segmentectomy
Source: Interdiscip Cardiovasc Thorac Surg. 2024 Jul 1;39(1):ivae125. doi: 10.1093/icvts/ivae125 (PMC11245319; doi:10.1093/icvts/ivae125)

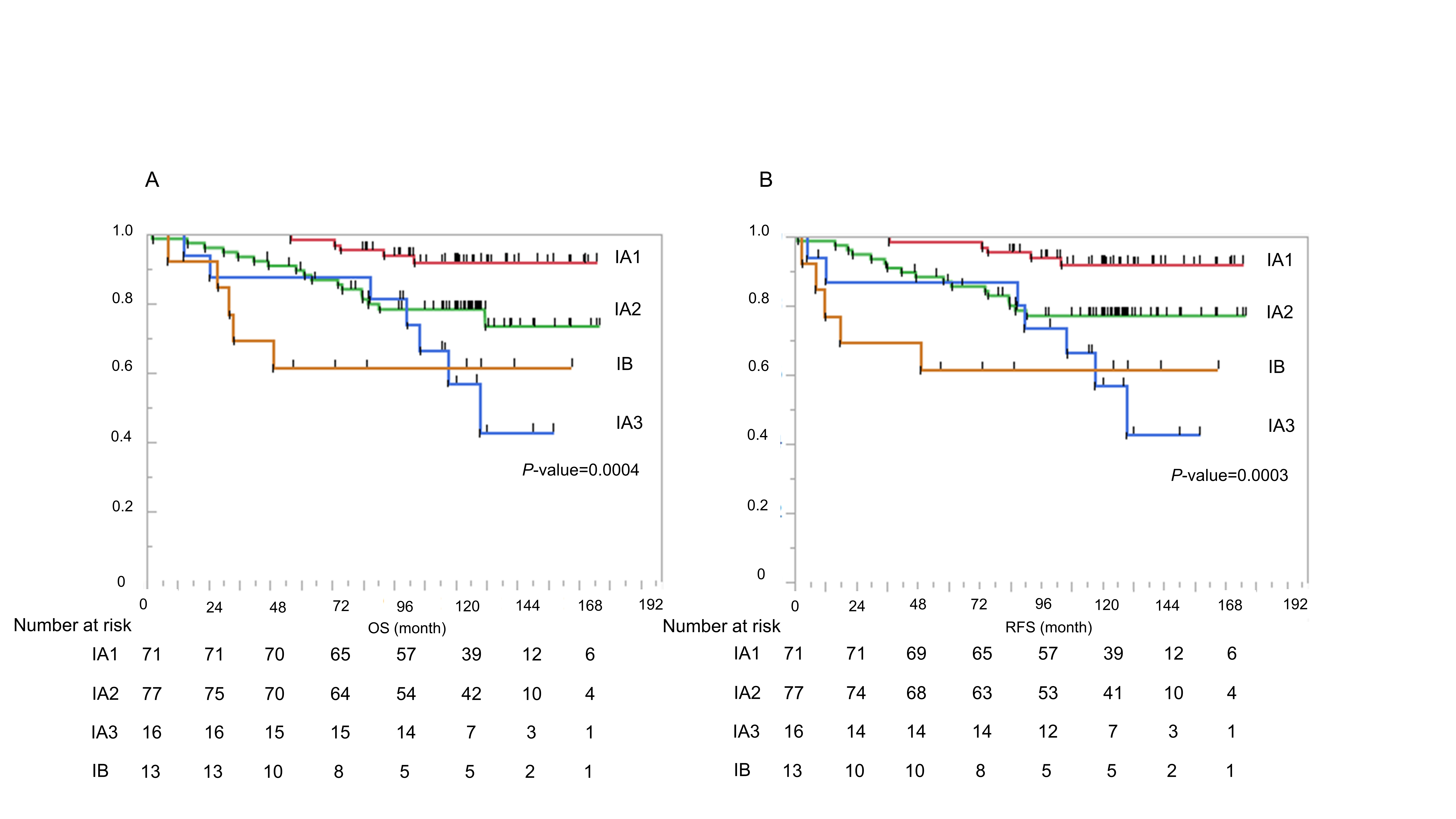

Supplement: ivae125_Supplementary_Data [file ivae125_supplementary_data.zip › Supplementary Figure4.tif]

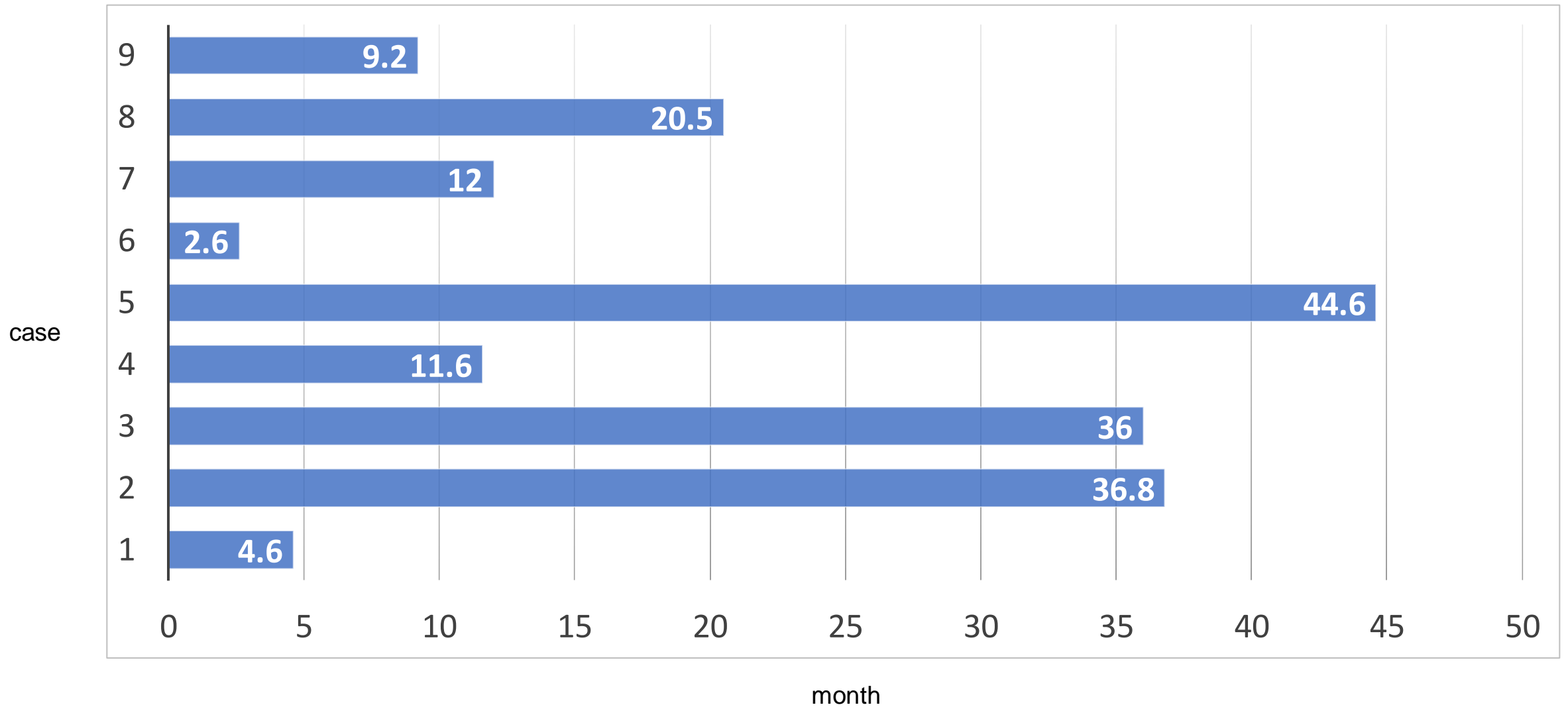

Supplementary Figure 1: Time to postsurgical recurrence

Supplement: ivae125_Supplementary_Data [file ivae125_supplementary_data.zip › Supplementary Fig1.pdf]

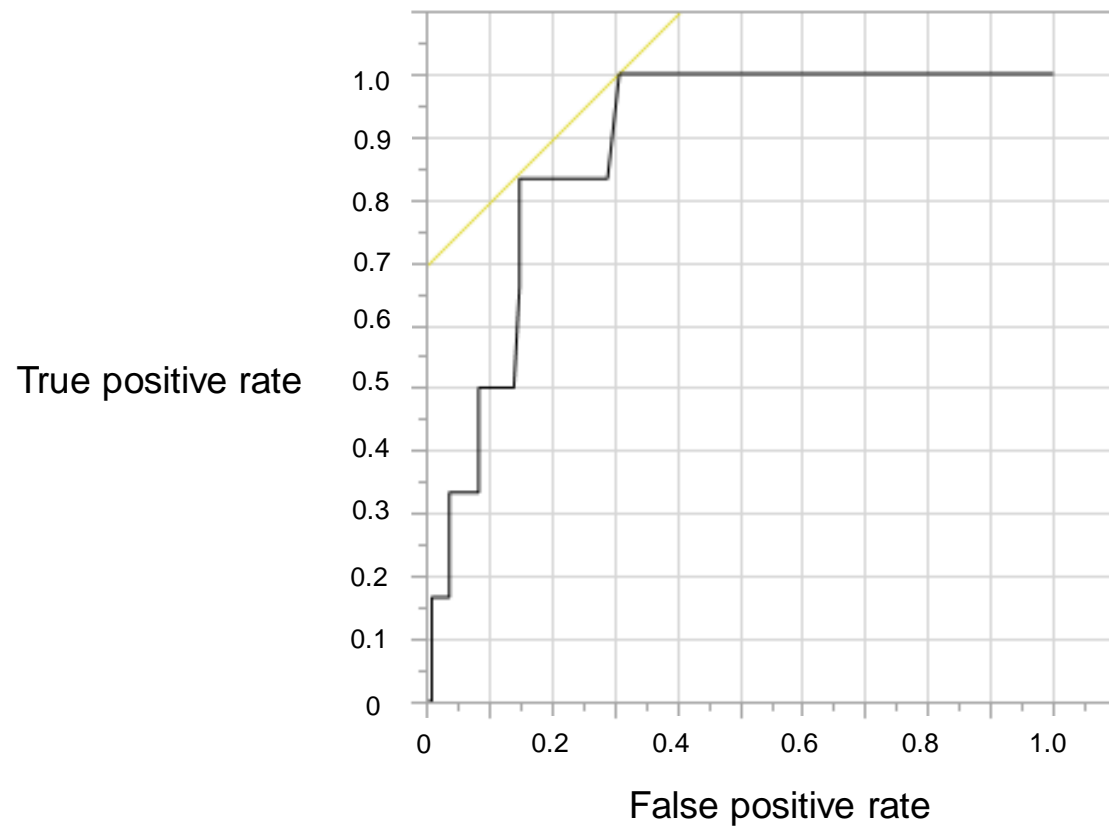

Supplementary Figure 2: Receiver operating characteristic (ROC) curves for tumor recurrence

Supplement: ivae125_Supplementary_Data [file ivae125_supplementary_data.zip › Supplementary Fig2.pdf]
